# Occurrence of Tin in Foods and Dietary Exposure Assessment in Zhejiang Province, China

**DOI:** 10.3390/foods15060982

**Published:** 2026-03-10

**Authors:** Shufeng Ye, Jiang Chen, Ronghua Zhang, Pinggu Wu, Dong Zhao, Xiaodong Pan, Jikai Wang, Hexiang Zhang, Xiaojuan Qi, Zijie Lu, Qing Ji, Biao Zhou

**Affiliations:** 1 School of Public Health, Ningbo University, Ningbo 315211, China; 17867970961@163.com; 2 Zhejiang Provincial Center for Disease Control and Prevention, Hangzhou 310051, China; jchen@cdc.zj.cn (J.C.); rhzhang@cdc.zj.cn (R.Z.); pgwu@cdc.zj.cn (P.W.); dzhao@cdc.zj.cn (D.Z.); xdpan@cdc.zj.cn (X.P.); jkwang@cdc.zj.cn (J.W.); hxzhang@cdc.zj.cn (H.Z.); xjqi@cdc.zj.cn (X.Q.); 130232023409@hmc.edu.cn (Z.L.); 3 School of Public Health, Hangzhou Medical College, Hangzhou 310013, China; huynquocotyee8423@gmail.com

**Keywords:** tin, pollution, dietary exposure, risk assessment

## Abstract

The food system of Zhejiang Province, a major coastal province in China, includes a wide variety of products, such as canned foods, aquatic products, vegetables, fruits, and tea, all of which may serve as potential sources of tin (Sn) exposure. However, no systematic study has assessed the distribution and dietary exposure risk of Sn across food categories in the province, and a compound-specific evaluation of organotin compounds is lacking. Therefore, we investigated the occurrence of Sn in commonly consumed foods and assessed dietary exposure risks among different age groups in Zhejiang Province. In total, 2014 samples from five major food categories—fresh vegetables, fresh fruits, tea, fresh aquatic products, and canned foods—were collected using a multistage stratified random sampling strategy. The Sn concentrations were measured using inductively coupled plasma mass spectrometry, with non-detection replaced by half the detection limit. Dietary intake data were derived from the 2015–2017 Nutrition and Health Surveillance using a 3-day, 24 h recall. The estimated daily intake and total hazard quotient (THQ) were calculated for age-specific risk assessments under multiple exposure scenarios. Fresh vegetables, fruits, tea, and most aquatic products had low Sn concentrations, whereas canned foods, particularly fruits, fungi, and meat products, had higher Sn concentrations. THQ values remained well below 1 across all food categories, indicating minimal health risks under typical consumption patterns, although lifetime exposure estimates suggested that canned foods could approach toxicological benchmarks earlier under high-consumption scenarios. A supplementary assessment of organotin compounds, which are highly toxic even at low fractions of total Sn, used a reverse dietary risk approach and probabilistic modeling. Canned foods and fresh aquatic products exhibited the lowest minimum conversion rates (0.16% and 0.37%, respectively), indicating that they are the most susceptible to organotin risk, whereas fresh fruits (7.77%) and tea (18.67%) required much higher proportions. Due to limited literature, further scenario- and probabilistic-based assessments focused on fresh aquatic products, revealing that typical exposure levels are generally safe, but children ≤ 6 years of age are the most vulnerable. Although overall Sn exposure is low, intake of highly processed foods, particularly canned products, should be limited in young children’s diets. These findings highlight that even small shifts in Sn speciation within high-risk food categories can lead to excessive tolerable daily intakes. This study provides a scientific reference for dietary Sn risk assessment and food safety management.

## 1. Introduction

Tin (Sn) is a post-transition metal with an atomic number of 50, naturally occurring in the Earth’s crust, primarily in the form of cassiterite (SnO_2_) and stannite (Cu_2_FeSnS_4_) [1]. Owing to their excellent corrosion resistance, ductility, and alloying properties, Sn and its compounds are widely used in various industrial sectors, including coatings, solders, batteries, plastics, and food packaging materials [2,3]. Over the past few decades, with the rapid development of electronics and new energy industries, global Sn consumption has increased markedly [4]. As of 2024, China is the largest producer of Sn worldwide, accounting for more than 30% of the global production [5].

Sn exists predominantly in inorganic and organotin forms, which differ substantially in their sources, physicochemical properties, and environmental fates, resulting in distinct entry pathways into the food chain. Inorganic Sn originates mainly from natural processes, such as mineral weathering, and anthropogenic activities, including mining, smelting, and the use of tin-containing materials in food contact and processing. It commonly occurs as Sn (II) or Sn (IV) species with low lipophilicity and a strong affinity for particulate matter, resulting in limited environmental mobility [2]. Consequently, inorganic Sn is preferentially retained in soils and sediments through adsorption to mineral surfaces and organic matter, which constrains its bioavailability and restricts its transfer into the food chain, primarily through direct contamination of plant-derived foods or incidental migration during food processing. Numerous studies have reported elevated Sn concentrations in industrial zones, mining areas, and estuarine regions, such as the Pearl River Delta in China [6], the Baltic Sea [7], and the coastal waters of Republic of Korea [8]. In contrast, organotin compounds are predominantly of anthropogenic origin and have been widely used as biocides, stabilizers, and industrial catalysts, although minor natural formations via microbial alkylation have been reported [9,10]. Owing to their pronounced lipophilicity, environmental persistence, and resistance to degradation, organotin compounds readily partition into biota- and organic-rich matrices, thereby facilitating bioaccumulation and trophic transfer [11]. These characteristics render organotin compounds more prone to enter the food chain and subsequently contribute to human dietary exposure, compared with inorganic Sn species [11].

Oral ingestion is the primary route of exposure to inorganic and organic compounds. Inorganic Sn is poorly absorbed (<5%); therefore, most ingested Sn is directly excreted via feces, and the small absorbed fraction is distributed mainly to the liver and kidneys, is metabolically inert, and is eliminated predominantly through urine [2]. In contrast, organotin compounds are efficiently absorbed owing to their lipophilicity [12,13] and are widely distributed in the soft tissues, including the liver, kidneys, central nervous system, and adipose tissue, where they can bind to proteins containing thiol groups [14,15]. Organotin compounds undergo hepatic dealkylation and are excreted via both urine and feces, partly through biliary excretion; however, their relatively long biological half-life results in tissue retention and prolonged elimination.

Toxicological studies in animals have indicated that high doses of inorganic Sn may induce gastrointestinal irritation and impair hepatic and renal functions, whereas organotin compounds can disrupt mitochondrial respiration and calcium homeostasis, leading to neurotoxicity, immunotoxicity, and endocrine-disrupting effects [16,17,18,19]. Of particular concern are the endocrine and developmental toxicities associated with organotin compounds. For instance, in Gannan lard poisoning, extremely elevated inorganic Sn concentrations were detected in pork fat (up to 664 mg/kg), which were associated with gastrointestinal symptoms and neurological manifestations in affected individuals [20]. Tributyltin (TBT) activates the nuclear receptors RXR and PPARγ, promoting adipocyte differentiation and functioning as an environmental obesogen [21,22]. Human studies have detected TBT and TPT in the maternal blood and placenta, with higher placental levels positively associated with cryptorchidism in newborns, indicating potential reproductive risks [23]. Moreover, prenatal exposure to organotin may result in reproductive toxicity, embryonic malformations, and early developmental defects such as cleft palate [24]. Collectively, these findings indicate that Sn exposure, particularly to organotin compounds, may have long-term adverse effects on human reproductive and developmental health.

In China, Sn has been detected in a wide range of foods including canned foods, aquatic products, fruits, and vegetables [25,26,27]. The Sn coating of food cans remains a major source of dietary Sn exposure, particularly when the inner protective lacquer is damaged or when acidic foods are stored for extended periods [28]. For example, Sn concentrations in six brands of tinned foods marketed in China ranged from 0.56 to 27.5 mg/kg, with the highest levels observed in canned yellow peaches, possibly owing to specific processing conditions [29]. Other studies have shown that the Sn content in fruit cans increases significantly with storage duration and repeated heating, ranging from 27.17 to 183.81 mg/kg after 3 months to 54.56–265.44 mg/kg after 12 months. Among aquatic products, the mean Sn concentration in shellfish (14.2 μg/kg) was markedly higher than that in fish [30]. Owing to their feeding habits, low mobility, and relatively long lifespan, mollusks tend to bioaccumulate Sn more readily than fishes. In vegetables, samples collected near mining areas exhibited elevated Sn levels (2.08 mg/kg in ferns), compared with those collected from non-mining regions (0.268 ± 0.004 mg/kg) [31]. However, data on Sn concentrations in fresh fruits in China are limited.

Similar findings have been reported in other countries. A study conducted in Italy revealed that the Sn concentrations in foods followed the order: preserved and canned fish > confectionery and pastries > meat products > aged cheese > other foods, indicating the potential migration of Sn from canned linings into food [32]. In Finland, dietary exposure to Sn through fish consumption was low, which is consistent with the results of Chinese studies [33]. Moreover, research from the United Kingdom has shown that the Sn concentration in citrus fruits (124 mg/kg) is markedly higher than that in other fruit types, suggesting species-specific differences in Sn accumulation capacity. Collectively, these studies indicate that industrial contamination, food processing techniques, and storage conditions can substantially influence human exposure to Sn, whereas food-contact materials remain the predominant source of inorganic Sn in the daily diet.

Although dietary exposure to Sn is generally low, its intake may exceed the safety threshold when consuming canned or contaminated foods. The European Food Safety Authority (EFSA) has established maximum levels of inorganic Sn at 100 mg/kg in canned beverages and 200 mg/kg in other canned foods. For infant formula and baby food, the maximum permissible concentration was set at 50 mg/kg [34].

Owing to the widespread presence of Sn in canned foods, aquatic products, and vegetables in China, several studies have analyzed the Sn concentrations in various foods and estimated the associated dietary exposure levels, yielding results consistent with international findings. Dai et al. [35] conducted a national dietary survey and reported that the daily dietary intake of Sn among Chinese residents was below the tolerable intake threshold. Hu et al. [36] found that dietary exposure to total Sn in fish and crustaceans raised in aquaculture ponds in the Zhejiang Province was below 0.045 mg/kg body weight, posing no evident health risks. However, domestic data on Sn exposure remain limited, particularly in vulnerable populations such as children and the elderly. Therefore, further dietary exposure assessments are warranted in regions with high consumption of canned foods and aquatic products to ensure food safety.

Several studies have evaluated dietary exposure to Sn in both domestic and international populations. The Joint FAO/WHO Expert Committee on Food Additives (JECFA) assessed inorganic Sn exposure across different population groups and concluded that exposure levels from canned foods and beverages are generally below the provisional tolerable weekly intake (PTWI), which is set at 14 mg/kg body weight per week [37]. The Agency for Toxic Substances and Disease Registry (ATSDR) has proposed a mid- to long-term oral minimal risk level (MRL) of 0.3 mg/kg bw/day based on a 13-week rat study with an NOAEL of 32 mg/kg/day, which can serve as a reference for chronic, non-carcinogenic exposure assessment [38]. Dietary monitoring in Italy indicated that Sn exposure among residents is well below this threshold, with fruits and vegetables being the major contributors [39]. A Malaysian study that assessed commercially available canned products reported a maximum estimated daily intake (EDI) of Sn at 0.03 mg/kg/day, which is far below the safety limit [40]. In Japan, dietary surveys among preschool children revealed an average daily Sn intake of approximately 10.6 µg/day, which is below the PTWI and does not indicate potential health risks [41].

In contrast, organotin compounds such as TBT, DBT, and TPT exhibit substantially higher toxicity and much lower safety thresholds. EFSA established a group tolerable daily intake (TDI) of 0.25 µg/kg bw/day for the sum of these species, whereas the U.S. EPA proposed an oral RfD of 0.3 µg/kg bw/day for tributyltin oxide, derived from immunotoxicity data [2,42]. These thresholds, which are several orders of magnitude lower than those of inorganic Sn, highlight the markedly higher chronic toxicity of organotin compounds.

Given the persistence, bioaccumulation potential, and multiple toxic effects of Sn and its organic derivatives, continuous monitoring of Sn contamination in food and environmental media is crucial. Regional investigations in Zhejiang Province have documented heavy metal contamination in aquaculture waters, sediments, and feed matrices. For example, Pb(II) concentrations ranged from 54 to 376 μg kg^−1^ in sediments and 14–94 μg kg^−1^ in feed, while levels in aquatic products were reported at 0.2–4.1 μg kg^−1^. In addition, Cd exhibited a relatively higher exceedance rate of 12.06%. Temporal monitoring data further indicated that Pb concentrations in agricultural soils increased overall from 2016 to 2020, with elevated levels predominantly observed in southern Zhejiang [36,43]. These findings suggest that rapid industrialization and intensive agricultural and aquaculture activities in Zhejiang may increase metal burdens in environmental compartments, which could subsequently enter the food chain and affect the safety of the local food supply. Although Sn concentrations in specific food items have been reported in Zhejiang Province [36], no comprehensive assessment has evaluated Sn distribution across major food categories or quantified population-level dietary exposure and health risks. Therefore, a systematic regional dietary risk assessment is warranted. As a major coastal province in China with intensive industrial and agricultural activities, Zhejiang’s food system includes a wide variety of products, such as canned foods, aquatic products, vegetables, fruits, and tea, all of which may serve as potential sources of Sn exposure. Building on our previous research on earth elements in aquatic products, vegetables, fruits, and tea [44], we conducted a comprehensive investigation of Sn levels in multiple food categories, including canned foods, aquatic products, vegetables, cereals, and fruits. Based on the measured concentrations, the THQ approach was applied to assess dietary exposure and potential health risks associated with Sn intake among residents of Zhejiang Province.

## 2. Materials and Methods

### 2.1. Sample Collection

Food sampling followed the formula below [45]:

$$N=\frac{Z^{2}\times \left[P\times \left(1-P\right)\right]}{d^{2}}$$

Using the standard sample size calculation formula, where *N* denotes the required number of samples, *Z* = 1.96 corresponds to a 95% confidence level, *P* = 0.5 represents the anticipated contamination proportion, and e = 0.10 indicates the allowable error, the theoretical minimum sample size was calculated to be 96.04. Considering that Sn contamination in food is generally lower and more variable than that of many other heavy metals, the target sample size was conservatively increased to 547 to ensure adequate statistical power and representativeness. A total of 2014 food samples were collected across the Zhejiang Province in 2018–2019 using a multistage stratified random sampling strategy. The sampling frame comprised 25 food types within five major categories: fresh vegetables, tea, fresh fruits, fresh aquatic products, and canned foods. Both bulk foods (including those repackaged on-site) and pre-packaged products were included. Samples were obtained from farmers’ markets, retail stores, street vendors, online stores, aquaculture sites, and capture fisheries to reflect actual market supply and dietary exposure of local residents. All samples were transported to the analytical laboratory immediately after collection, sealed to avoid contamination, and frozen until Sn determination.

Detailed information on the geographic origin and distribution of the collected food samples is provided in Table S1. The provincial origin of canned food samples is summarized in Table S2. The distribution of packaging types and sampling site categories across food groups is presented in Table S3.

### 2.2. Methods

#### 2.2.1. Determination of Sn Content

All participating laboratories performed the analyses under unified operational protocols, and all staff involved in laboratory testing and data management received formal training prior to sample processing. Quantification of Sn in food matrices was conducted using inductively coupled plasma mass spectrometry (ICP-MS), following the technical requirements outlined in the National Manual for Risk Monitoring of Food Contaminants and Hazardous Factors [46]. Samples were dried prior to analysis, and all Sn concentrations are expressed on a dry weight basis. For data treatment, non-detection results were handled according to the WHO guidelines for evaluating trace-level contaminants in food [47], where values below the detection limit were substituted with one-half of the LOD. The method detection limit (mg/kg) for Sn was 0.004 mg/kg for fresh vegetables, fresh fruits, fresh aquatic products, and canned foods, whereas tea samples had a slightly higher detection limit of 0.008 mg/kg.

#### 2.2.2. Resident Consumption Survey

In this study, dietary consumption information was derived from the 2015 to 2017 Nutrition and Health Surveillance conducted in the Zhejiang Province. Surveillance was conducted in 10 counties (cities or districts), and eligible participants were those who had resided locally for at least 6 months. A multistage stratified random sampling strategy was applied at the household level, and all household members were invited to participate. Informed consent was obtained from all respondents, and their guardians provided consent for minors. The survey included approximately 10,000 participants. The dietary intake of various food categories, including fresh vegetables, tea, fresh fruits, fresh aquatic products, and canned foods, was assessed using a 3-day, 24 h dietary recall method, capturing the usual consumption patterns across different age groups. These data were subsequently used to estimate the daily intake of Sn from each food category for the exposure assessment.

#### 2.2.3. Quality Control

When measuring pollutant concentrations, all sample analyses were conducted in strict accordance with the relevant national standard operating procedures. Both intralaboratory and interlaboratory quality control measures were implemented throughout the testing process. Certified reference materials were used to verify instrument performance before, during, and after the analytical runs. For instruments lacking appropriate interference–removal modes, interference-correction equations were applied to adjust the measured values. In addition, two procedural blanks were analyzed alongside each batch of samples to ensure the accuracy and reliability of the obtained results.

#### 2.2.4. Exposure Assessment of Consumers

The risk of Sn exposure for other food consumers was calculated using this formula:

$$EDI=\frac{Cv\times CR}{BW\times 1000}$$

where *EDI* is the daily intake of Sn in vegetables per kilogram of body weight (mg/kg BW), which is the concentration of Sn in fresh vegetables, tea, fresh fruits, fresh aquatic products, and canned foods (mg/kg) calculated based on wet weight; *Cv* is the concentration of total Sn in each food; *CR* is the average daily consumption (g/day); and *BW* is the body weight (kg) of each individual.

#### 2.2.5. Health Risk Assessment of Sn

To evaluate the cumulative health risk associated with dietary exposure to total Sn, we calculated the total hazard quotient (THQ) using the following formula:

$$THQ=\sum_{i=1}^{n}{HQ}_{i}$$

where ${HQ}_{i}$ represents the hazard quotient for Sn originating from each food category and is calculated as:

$${HQ}_{i}=\frac{{EDI}_{i}}{RfD}$$

where ${EDI}_{i}$ is the estimated daily intake of Sn from a given food category (mg/kg bw/day), and *RfD* is the reference dose for inorganic Sn. Following the recommendation of the Agency for Toxic Substances and Disease Registry (ATSDR), an oral minimal risk level (MRL) of 0.3 mg/kg BW/day for chronic-duration exposure was adopted as the toxicological benchmark for risk characterization [38]. Although this value was derived for inorganic Sn, it was conservatively applied to the total Sn exposure for the screening-level assessment.

If *THQ* > 1, the potential hazard of Sn in food should be considered with caution, whereas *THQ* < 1 indicates that the health risk of Sn in food is within an acceptable range.

#### 2.2.6. Dietary Exposure Assessment of Organotin Compounds

In this study, compound-specific quantification of organotin species (TBT, DBT, TPT, and DOT) by LC–MS/MS or GC–MS was not performed. Total Sn concentrations were determined in food samples, and dietary exposure to organotin compounds was estimated only for fresh aquatic products based on the proportion of organotins relative to total Sn. Owing to the lack of compound-specific monitoring data, a conservative screening approach was developed by applying a literature-derived minimum proportion of organotin relative to total Sn (5.83%) reported for mollusks [48]. This proportion was used as a protective benchmark to evaluate whether dietary organotin exposure from aquatic products could plausibly exceed toxicological reference values under worst-case conditions. The TDI for organotins is 0.25 µg/kg body weight/day (as organotin compounds) [49], which corresponds to approximately 0.10 µg-Sn/kg body weight/day when expressed as Sn, based on the molecular weight ratio between Sn and organotin compounds [50].

EDI of organotins were calculated using the following equation:

$${EDI}_{organotin}=Food\;Consumption\times Total\;Sn\;Concentration\times 0.0583$$

where 0.0583 represents the organotin fraction of the total Sn (5.83%). Subsequently, *THQ* was computed for each food category by dividing the *EDI* by the organotin-specific *TDI*.

$${THQ}_{organotin}=\sum \frac{{EDI}_{organotin}}{{TDI}_{organotin}}$$

If ${THQ}_{organotin}$ > 1, the potential hazard of organotin for food should be considered with caution, whereas ${THQ}_{organotin}$ < 1 indicates that the health risk of organotin for food is within an acceptable range.

#### 2.2.7. Probabilistic Risk Assessment

Dietary exposure to organotin was probabilistically assessed using a Monte Carlo simulation with 10,000 iterations. Concentrations for each food category were sampled from the observed data, whereas the consumption of non-canned foods was obtained from 24 h dietary recalls. The exposure per iteration was calculated as follows: concentration × consumption ÷ body weight, using an average body weight of 57 kg derived from the Zhejiang provincial dietary consumption survey. The total and category-specific exposures, as well as the probabilities of exceeding the TDI, were derived from the simulated distributions.

#### 2.2.8. Statistical Analysis

Data entry and processing were conducted using Excel 2010, and statistical analyses were performed using R version 4.3.3. Statistical significance was set at *p* < 0.05. The distributions of Sn concentrations and dietary consumption across different food categories were not normal (*p* < 0.05); hence, descriptive percentiles [50th percentile (P50) and 95th percentile (P95)] were employed to summarize the exposure differences among the foods and population subgroups.

For dietary exposure estimation, high-percentile consumption data from the survey were considered. P95 of intake was used to represent individuals with high consumption, whereas Sn concentrations obtained via mass spectrometry were highly precise, and the maximum measured values were applied to represent high-contamination scenarios. Four exposure scenarios were defined as follows: A, P50 consumption × P50 Sn concentration; B, P95 consumption × P50 Sn concentration; C, P50 consumption × P95 Sn concentration; and D, P95 consumption × P95 Sn concentration. Sn intake was calculated for each age group (≤6, 7–12, 13–17, 18–59, and ≥60 years).

## 3. Results

### 3.1. Distribution Characteristics of Sn in Foods

A total of 2014 food samples were included in the analysis (Table 1): fresh vegetables (*n* = 673), tea (*n* = 378), fresh aquatic products (*n* = 392), fresh fruits (*n* = 133), and canned foods (*n* = 438). These samples covered the major food categories relevant to the dietary Sn exposure assessment. The detection frequency of Sn varied across the categories: fresh vegetables and fruits generally exhibited low detection rates, aquatic products showed moderate detection rates, and tea samples demonstrated consistently high detection frequencies. Canned foods also showed relatively high detection rates, which is consistent with the recognized potential for Sn migration from can-linings during processing and storage. Across all food categories, none of the samples exceeded the national maximum allowable Sn limits, with all canned foods remaining below 100 mg/kg and all other food categories remaining below 200 mg/kg.

The Sn concentration varied markedly among the different food categories. Fresh vegetables exhibited generally low levels, mostly ranging from 0.01 to 0.06 mg/kg, with tuber and root vegetables showing the highest mean concentration (0.06 mg/kg, maximum 4.53 mg/kg) and leafy and solanaceous vegetables at 0.04 mg/kg. Tea contained slightly higher concentrations, ranging from 0.08 to 0.13 mg/kg, with oolong tea showing the highest mean value (0.13 mg/kg). Among aquatic products, fish had the highest Sn levels (0.26 mg/kg, maximum 10.30 mg/kg), substantially exceeding those in crustaceans and mollusks (0.01–0.02 mg/kg). Canned foods displayed the most pronounced differences, with canned fruits showing the highest mean concentration (12.67 mg/kg, maximum 134 mg/kg), followed by canned vegetables and canned meat at 1.05 and 0.96 mg/kg, respectively, while other canned items remained below 2 mg/kg. Overall, Sn concentrations followed a clear gradient: lowest in fresh vegetables, intermediate in tea and aquatic products, and highest in canned food.

### 3.2. Daily Consumption of Sn Exposure Among Consumers

Daily food consumption among residents of Zhejiang varied notably according to age and food category (Table 2). The median intake ranged from 35 to 100 g/day of fresh vegetables, 80 to 160 g/day of fruits, 50 to 80 g/day of fresh aquatic products, 0 to 6 g/day of tea, and 0 to 300 g/day of canned foods, with cereal-based canned foods and canned meat at the higher end of this range. Statistically significant differences in consumption across age groups were observed for most food categories (Kruskal–Wallis test, *p* < 0.05), reflecting clear age-dependent dietary patterns.

### 3.3. Dietary Exposure Risks of Inorganic Sn

#### Dietary Exposure Risks of Inorganic Tin

**Figure 1:**
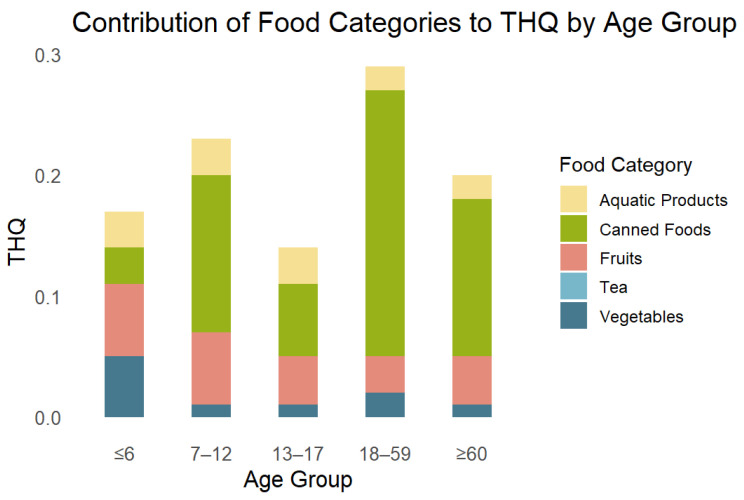
Contribution of food categories to THQ by age group (light blue box—tea contribution is almost zero).

The estimated dietary exposure to Sn among Zhejiang residents differed across food categories and age groups (Table 3 and Figure 1). Under typical consumption levels (P50 × P50), the exposure to most foods is generally below 0.01 mg/kg·bw/day. Higher contributions were observed from canned foods, particularly canned meat, cereal-based canned foods, and canned aquatic products, under high-consumption and high-concentration scenarios (P95 × P95), with the highest intake reaching 11.45 mg/kg·bw/day for canned meat in children ≤ 6 years. Among fresh foods, tuber and root vegetables, solanaceous vegetables, and mollusks contributed most substantially, whereas pome fruits were the main contributors.

Age-specific patterns are also observed. Children ≤ 6 years showed relatively higher exposure from canned meat and pome fruits, reflecting both consumption habits and body weight. Adolescents (7–17 years) exhibit moderate exposure to vegetables and canned products. Adults (18–59 years) had higher intakes from vegetables, cereal-based canned foods, and mollusks, while elderly residents (≥60 years) displayed moderate exposure across most food categories, with slightly higher contributions from canned foods. Overall, the sum of estimated daily intakes (ΣEDI) ranged from 5.48 to 65.29 µg/kg BW/day, and all target hazard quotients (THQ) remained below 1, indicating that dietary Sn exposure in this population is within safe levels.

### 3.4. Dietary Exposure Risks of Organic Sn

#### 3.4.1. Minimum Organotin Conversion Proportions for Dietary Toxicity Thresholds

**Figure 2:**
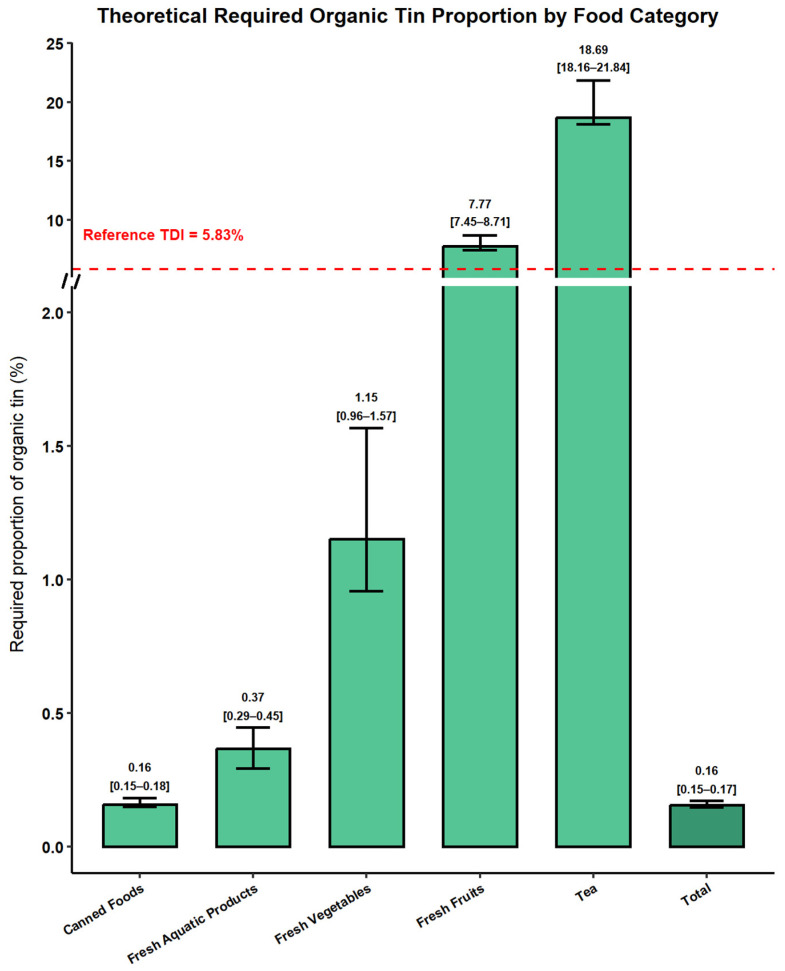
Minimum Proportion of Inorganic Tin Converted to Organotin under a Theoretical Estimation Scenario.

Given the high toxicity of organotin compounds, a highly conservative reverse exposure assessment was conducted based on the organotin reference daily intake (*RDI*) and existing data on concentrations and consumption (Figure 2). This analysis aimed to determine the minimum proportion of total tin that would need to exist as organotin in different food categories to reach the toxicological threshold. Under an extremely conservative scenario (P0.5, the 0.5th percentile of the Monte Carlo-simulated exposure distribution), a reverse exposure assessment was conducted to estimate the minimum proportion of inorganic Sn that must be converted to organotin to reach the toxicological threshold. The required conversion proportions varied substantially across the food categories (Figure 2). The lowest proportions were observed for canned foods (0.16%, 95% CI: 0.15–0.18%) and fresh aquatic products (0.37%, 95% CI: 0.29–0.45%), followed by fresh vegetables (1.15%, 95% CI: 0.96–1.57%). In contrast, markedly higher proportions were required for fresh fruits (7.77%, 95% CI: 7.45–8.75%) and tea (18.67%, 95% CI: 18.14–21.93%). For the overall diet, the required proportion was 0.16% (95% CI: 0.15–0.17%), indicating that even under this highly conservative assumption, relatively small increases in organotin formation at the dietary level could lead to an exceedance of the TDI.

#### 3.4.2. Scenario-Based Screening of Dietary Organotin Exposure

Based on the existing data on organotin fractions in total tin concentrations, the dietary exposure to organotins from fresh aquatic products among Zhejiang residents was assessed, with variations noted across age groups and exposure scenarios (Table 4). In Scenario A, which considers median consumption and contamination levels (P50 × P50), the ∑EDI values were low, ranging from 0.02 µg/kg BW/day in adults (18–59 years) to 0.04 µg/kg BW/day in children aged ≤6 years. The corresponding THQ values were between 0.2 and 0.4, indicating that the exposure levels were well below the reference dose (RDF = 0.1 µg/kg BW/day). In Scenario B (high consumption, median contamination, P95 × P50), the ∑EDI values moderately increased across all age groups to 0.07–0.15 µg/kg BW/day, with THQ values rising to 0.7–1.5, highlighting that younger children remained the most exposed group. Under Scenario C (median consumption, high contamination, P50 × P95), the ∑EDI values increased substantially to 0.70–1.60 µg/kg BW/day, with THQ values of 7.0–16.0, indicating a marked risk in younger age groups. Scenario D (high consumption and high contamination, P95 × P95) resulted in the highest estimates, with ∑EDI reaching 2.03–3.68 µg/kg BW/day and THQ values of 20.3–36.8, further highlighting the highest absolute exposure in children aged ≤6 years.

#### 3.4.3. Monte Carlo Simulation of Dietary Organotin Exposure

In scenario B, the total dietary exposure in children aged ≤6 years exceeded the RDF, and in high-exposure Scenarios C and D, all age groups experienced exposure levels well above the RDF (Table 5). Consequently, a refined Monte Carlo-based dietary exposure assessment was performed. The findings revealed that organotin exposure from fresh aquatic products varied across age groups but generally approached or exceeded the tolerable daily intake (TDI) of 0.1 µg/kg BW/day). Monte Carlo simulations revealed the percentiles at which the TDI was reached as follows: 69.3% (95% CI: 68.4–70.2%) for ≤6 years, 67.7% (95% CI: 66.8–68.6%) for 7–12 years, 65.8% (95% CI: 64.8–66.7%) for 13–17 years, 65.0% (95% CI: 64.2–65.9%) for 18–59 years, 65.6% (95% CI: 64.6–66.6%) for ≥60 years, and 66.3% (95% CI: 65.4–67.3%) for the total population. These findings suggest that while the average exposure remains below toxicological thresholds, a substantial proportion of individuals with high consumption, especially young children (≤6 years), may reach or exceed the TDI, underscoring their increased vulnerability to dietary organotin exposure from fresh aquatic products.

## 4. Discussion

The Sn concentrations showed clear differences across food categories. Those in fresh vegetables and fruits were generally low, with most samples exhibiting P50 values close to or equal to zero, suggesting limited background exposure. Among fresh vegetables, tuber and root vegetables contain higher Sn levels than other types, likely because of prolonged direct contact with soil, which facilitates the adsorption of soil particles and associated Sn on the surface [51]. Although specific studies comparing the Sn content of roots and leafy vegetables are lacking, prior research on metals such as Cd and Pb has shown that root vegetables tend to retain metals through root sequestration and limited xylem transport [52,53]. Fresh fruits exhibited even lower Sn levels, with a majority of samples having a P50 of 0 and P95 generally below 0.03 mg/kg, indicating a minimal exposure risk from fruit consumption.

Regarding tea, detected Sn concentrations were low (green tea 0.03 mg/kg, black tea 0.03 mg/kg, red tea 0.04 mg/kg, oolong tea 0.03 mg/kg), lower than previously reported values; for example, tea from the Sichuan Province contained 0.07 mg/kg [54], and the Fujian Province teas averaged 0.12 ± 0.077 mg/kg [55]. The higher detection rates observed in these studies may be attributed to the concentration of metals during the dehydration of the dry tea samples. In fresh aquatic products, fish samples exhibited substantial variability, with maximum concentrations reaching 10.30 mg/kg, which were significantly higher than those observed in crustaceans and mollusks. However, the detection rate in fish was only 15% and the median value was near zero, indicating that elevated concentrations were driven by a limited number of samples. Crustaceans and mollusks generally contained low Sn levels (average ≤ 0.02 mg/kg) with a relatively narrow distribution, suggesting stable exposure levels.

Both the detection frequency and Sn concentration were the highest in canned foods. The median Sn concentration in canned fruits reached 0.15 mg/kg, which is consistent with findings from France [56] and the United Kingdom [57], where the dietary intake of Sn in adults largely depended on foods stored in Sn cans (98%). This is likely attributable to the canning process; when the internal coating of the can is damaged or absent, Sn can leach under high-temperature sterilization and acidic conditions. In this study, Sn concentrations in canned fruits were notably higher than in other canned foods; low-acidity vegetable cans (0.12 mg/kg) and meat cans (0.03 mg/kg) contained lower levels. Experimental studies have shown that acidic canned products generally contain higher Sn levels than non-acidic cans or fresh foods [55,58], likely because the acidic matrix accelerates the migration of Sn from the interior, particularly during high-temperature processing and prolonged storage [59]. Sn concentrations in canned fish sold in Iran ranged from 0.036 to 0.480 mg/kg [60], and a study in the Liaoning Province reported 0.15–6.34 mg/kg in fish cans [61]. In this study, Sn levels in canned fish were relatively low at 0.12 mg/kg, possibly due to shorter storage durations.

Food consumption varied markedly across the age groups. The intake of fresh vegetables, fruits, and aquatic products generally increased from childhood to adulthood, with adults and older adults showing the highest P50 and P95 values across most categories. Tea consumption was negligible in children and adolescents, but became more prominent in adults, especially green and oolong tea. Canned food consumption remained low overall, although the high-percentile intake among some age groups indicated an occasional peak exposure.

Compared with previous reports from China [35] and other regions [39,41], the estimated dietary exposure to Sn among Zhejiang residents was generally consistent with the low levels reported in adults and children. Similar to findings in European populations [56], canned foods, particularly canned meat, cereal-based products, and aquatic items, emerged as the primary contributors under high consumption scenarios, whereas fresh vegetables, fruits, and mollusks contributed modestly. Adults aged 18–59 years contributed the most to the total dietary Sn exposure, whereas children and adolescents, despite having a lower absolute intake, remained relatively sensitive because of higher intake-to-body-weight ratios [41], with older adults showing intermediate levels. Overall, the THQ values remained well below 1, indicating minimal health risks, which is consistent with previous national and international assessments [62]. However, under the highest exposure scenario (P95 × P95), adults aged 18–59 years reached a THQ of 0.22, indicating that high-consumption subgroups could experience relatively elevated exposure. Nonetheless, high-percentile scenarios underscore the importance of monitoring canned food products because occasional high-exposure batches may disproportionately affect sensitive subpopulations. Although the current diet does not indicate a significant tin-related health risk, highly processed foods, particularly canned products, should be limited in the diets of young children as a precautionary measure.

Because the total Sn measurements do not differentiate between toxic organotin species and less toxic inorganic forms, a reverse dietary risk assessment was conducted under an extremely conservative exposure scenario (P0.5) to determine the minimum proportion of inorganic Sn required to reach a toxicological threshold. Instead of relying on fixed organotin fractions from the literature, threshold proportions were back-calculated from the lower end of the population-level exposure distribution. The findings indicated that the minimum conversion proportions were lowest for canned foods (0.16%, 95% CI: 0.15–0.18%) and fresh aquatic products (0.37%, 95% CI: 0.29–0.45%), whereas substantially higher proportions were required for fresh fruits (7.77%) and tea (18.67%), highlighting the differences in consumption patterns and contamination levels across different food categories. At the total diet level, the threshold proportion was estimated to be 0.16%, comparable to that derived under typical exposure scenarios, suggesting that minor changes in Sn speciation within high-risk food categories could result in exceeding the tolerable daily intake. These results demonstrate that identifying high-risk foods is insensitive to exposure percentile assumptions and offers a conservative yet robust scientific basis for future organotin toxicity assessments. In future tests, if the proportion of organotin to total Sn approaches or exceeds the threshold levels established under this conservative scenario, the corresponding toxicological risk threshold may be exceeded.

Reverse risk assessments indicated that only a small fraction of the total Sn needs to be converted to organotin species for canned foods, fresh aquatic products, and vegetables to reach the toxicological threshold. However, most quantitative organotin speciation data in the literature focus on aquatic organisms, with crustaceans showing the lowest documented conversion ratio (5.83%) [48], while data for canned foods and vegetables are limited. Accordingly, this minimum ratio was conservatively applied to fresh aquatic products to assess potential organotin-related health risks. Scenario-based assessment of fresh aquatic products showed that dietary exposure to organotin is strongly influenced by both consumption patterns and contamination levels. Under typical conditions (Scenario A), exposure was well below the reference dose (RDF = 0.1 µg/kg BW/day) across all age groups, with THQ values of <1. Higher consumption (Scenario B) or elevated contamination (Scenario C) led to moderate exceedances, particularly in children ≤ 6 years of age, highlighting their greater vulnerability to organotin intake. Scenario D, which combined high consumption and high contamination, led to the greatest exposure and THQ values, demonstrating that simultaneous extreme conditions for both factors greatly elevated the risk. These findings emphasize that fresh aquatic products can significantly contribute to dietary organotin exposure under adverse conditions, consistent with previous studies [33,43,63,64], and emphasize the need for targeted monitoring and mitigation strategies for high-risk age groups (≤6 years).

In contrast to deterministic point estimates, Monte Carlo simulations offer a more comprehensive probabilistic assessment by better capturing variability in contamination levels and consumption patterns [65]. For fresh aquatic products, children aged ≤6 years reached the TDI at the 69.3 percentile (95% CI: 68.4–70.2%), which was higher than that of older age groups (65.0–67.7%), indicating that younger children face a relatively greater risk. This increased exposure is primarily attributed to their higher food intake relative to body weight, which increases the dietary exposure per kilogram in this sensitive subpopulation [46]. A similar Monte Carlo–based probabilistic assessment of butyl- and phenyl-tin exposure from fish and seafood in the Taiwanese population revealed higher health risks, with mean hazard indices (HIs) exceeding 1 in children < 12 years of age and the 95% upper confidence limits of HIs exceeding 1 in most age and sex groups, highlighting a wider population-level concern [64]. In comparison, the lower probabilistic exposure levels observed in this study imply a reduced dietary organotin burden from aquatic products among residents of Zhejiang, potentially reflecting improved organotin emission controls in recent years. However, the consistent identification of young children as the most vulnerable group underscores the need for continued probabilistic risk assessment and focused surveillance of organotin in aquatic food products.

This study had some limitations. Only a limited range of food types was analyzed, and the consumption data (2015–2017) and Sn concentration data (2018–2019) were collected during different periods, which may have introduced uncertainty into the exposure assessment. Although no major dietary transitions were reported during this interval, potential changes in dietary patterns, food processing practices, or food packaging materials (particularly the use of canned products) could influence population-level exposure estimates. Therefore, the temporal mismatch between dietary and concentration data may affect the representativeness of the results. Furthermore, the absence of established toxicological reference values for specific Sn species, the lack of direct measurements of organotin compounds in food, and the limited number of studies addressing both total Sn and organotin speciation in foods constrain the accuracy of the risk assessment.

Future studies should provide more comprehensive evaluations of dietary Sn intake, especially in high-consumption subgroups that may accumulate higher Sn exposure over time. Continuous monitoring of canned food products and updated consumption surveys are needed, alongside targeted measurements of organotin species in different food categories and investigations of the long-term health effects of chronic low-level Sn exposure.

## 5. Conclusions

In summary, dietary exposure to total Sn among Zhejiang residents was generally low, with most fresh vegetables, fruits, and tea containing minimal levels, and fresh aquatic products mostly low except for occasional high concentrations in fish; canned foods showed the highest Sn concentrations, particularly acidic canned fruits (median 0.15 mg/kg), due to potential leaching from can coatings. Although overall exposure to total Sn is low, organotin compounds are extremely toxic and require only a small fraction of total Sn to reach toxicological thresholds. A reverse dietary risk assessment revealed that the minimum conversion rate was lowest for canned foods (0.16%) and fresh aquatic products (0.37%), whereas fresh fruits and tea had higher rates of 7.77% and 18.67%, respectively. Due to limited literature on organotin species in canned foods, further scenario- and probabilistic-based evaluations focused on fresh aquatic products, showed that typical exposure levels are generally safe, but children ≤ 6 years old and high-consumption subgroups are the most vulnerable. Therefore, even though overall total Sn exposure does not exceed safety thresholds, highly processed foods (especially canned products) should be avoided in the diets of young children to reduce potential exposure to Sn. These findings highlight that, while total Sn poses minimal risk, organotin intake, particularly from fresh aquatic products, warrants targeted monitoring to safeguard sensitive populations.

## Figures and Tables

**Table 1:** Concentration of Sn in each food (mg/kg, dry weight).

Category	Sample Size	Detect (*n*) %	Concentration (mg/kg)					
			Mean	P50	P95	IQR	Min	Max
Fresh Vegetables								
Solanaceous Vegetables	32	(11) 34	0.04	0.00	0.27	0.01	0.00	0.34
Cruciferous Vegetables	40	(12) 30	0.01	0.00	0.03	0.00	0.00	0.04
Cucurbit Vegetables	24	(4) 17	0.01	0.00	0.04	0.00	0.00	0.04
Tuber and Root Vegetables	239	(72) 30	0.06	0.00	0.33	0.01	0.00	4.53
Aquatic Vegetables	199	(64) 32	0.01	0.01	0.04	0.01	0.00	0.10
Leafy Vegetables	139	(35) 25	0.04	0.00	0.21	0.00	0.00	1.14
Tea								
Green Tea	268	(204) 76	0.08	0.03	0.37	0.08	0.00	1.30
Black Tea	55	(45) 82	0.08	0.04	0.31	0.06	0.00	0.57
Oolong Tea	31	(25) 81	0.13	0.03	0.31	0.09	0.00	2.02
Dark Tea	24	(23) 96	0.08	0.03	0.49	0.03	0.00	0.57
Fresh Aquatic Products								
Fish	175	(26) 15	0.26	0.01	0.50	0.15	0.00	10.30
Crustaceans	157	(90) 57	0.02	0.01	0.10	0.01	0.00	0.40
Mollusks	60	(26) 43	0.01	0.00	0.03	0.00	0.00	0.50
Fresh Fruits								
Citrus Fruits	8	(1) 13	0.00	0.00	0.01	0.00	0.00	0.02
Melons and Gourd Fruits	9	(3) 33	0.01	0.00	0.02	0.00	0.00	0.02
Stone Fruits	19	(7) 37	0.00	0.00	0.01	0.00	0.00	0.02
Berries and Other Small Fruits	36	(3) 8	0.00	0.00	0.00	0.00	0.00	0.02
Tropical and Subtropical Fruits	38	(6) 16	0.00	0.00	0.01	0.00	0.00	0.03
Pome Fruits	23	(9) 39	0.02	0.00	0.03	0.01	0.00	0.24
Canned Foods								
Cereal-based Canned Foods	52	(27) 52	0.04	0.01	0.21	0.04	0.00	0.37
Canned Vegetables and Vegetable Products	57	(38) 67	1.05	0.12	5.13	0.23	0.00	19.80
Canned Fruits and Fruit Products	82	(65) 79	12.67	0.15	80.73	5.79	0.00	134.00
Canned Meat and Meat Products	71	(47) 66	0.96	0.03	3.42	0.10	0.00	23.70
Canned Aquatic Products	93	(62) 67	0.12	0.02	0.62	0.10	0.00	1.51
Canned Edible Fungi and Fungal Product	83	(60) 73	1.61	0.06	0.48	0.12	0.00	38.90

**Table 2:** Food consumption and body weight of Zhejiang residents by age group.

Category	P50 (g/Day)					P95 (g/Day)				
	≤6	7–12	13–17	18–59	≥60	≤6	7–12	13–17	18–59	≥60
Fresh Vegetables										
Solanaceous Vegetables	50	60	80	96	100	120	150	150	180	200
Cruciferous Vegetables	50	80	100	200	105	50	130	150	200	200
Cucurbit Vegetables	50	60	80	100	100	131.7	150	174.4	200	200
Tuber and Root Vegetables	43.5	60	79	100	100	100	150	148	200	200
Aquatic Vegetables	35	50	77.5	80	82	101	140	146.5	160	181.2
Leafy Vegetables	50	80	100	100	100	120	150	180	200	200
Tea										
Green Tea	0	1	3	3	4	0	1	5.25	10	11.75
Black Tea	0	3	3	3	2	0	3	3	7.45	6.1
Oolong Tea	0	0	0	3	6	0	0	0	10	6
Dark Tea	0	0	0	3	5	0	0	0	1.5	2
Fresh Aquatic Products										
Fish	50	50	70	80	70	100	150	150	200	200
Crustaceans	50	50	60	60	52	120	150	200	175	150
Mollusks	60	60	80	80	70	400	350	400	500	500
Fresh Fruits										
Citrus Fruits	80	100	106	110	200	180	180	200	200	200
Melons and Gourd Fruits	120	140	180	180	180	250	300	250	380	350
Stone Fruits	100	123.5	150	150	150	230	240	250	258	258
Berries and Other Small Fruits	80	100	100	100	105	200	200	200	240	250
Tropical and Subtropical Fruits	96	100	115	120	120	200	216.5	250	250	250
Pome Fruits	120	148	160	160	155	225	240	250	258	258
Canned Foods										
Cereal-based Canned Foods	0	0	300	150	355	0	0	300	360	465
Canned Vegetables and Vegetable Products	0	20	0	20	15	0	20	0	20	19
Canned Fruits and Fruit Products	0	0	0	40	0	0	0	0	40	0
Canned Meat and Meat Products	10	13.3	20	20	13.3	66.7	100	123.3	66.7	66.7
Canned Aquatic Products	16.65	22.2	33.3	33.3	22.2	93.3	139.95	186.7	93.3	93.3
Canned Edible Fungi and Fungal Product	0	20	0	40	35	0	29	0	46	35
Body Weight (kg)	19.9	33.7	52.3	62.1	60.4	19.9	33.7	52.3	62.1	60.4

**Table 3:** Estimated dietary exposure risk per food item in residents.

Category	≤6	7–12	13–17	18–59	≥60	≤6	7–12	13–17	18–59	≥60
	A: P50 × P50					B: P95 × P50				
Fresh Vegetables										
Solanaceous Vegetables	0.01	0.00	0.00	0.00	0.00	0.01	0.01	0.01	0.01	0.01
Cruciferous Vegetables	0.01	0.00	0.00	0.01	0.00	0.01	0.01	0.01	0.01	0.01
Cucurbit Vegetables	0.01	0.00	0.00	0.00	0.00	0.01	0.01	0.01	0.01	0.01
Tuber and Root Vegetables	0.00	0.00	0.00	0.00	0.00	0.01	0.01	0.01	0.01	0.01
Aquatic Vegetables	0.01	0.01	0.01	0.01	0.01	0.03	0.02	0.01	0.01	0.02
Leafy Vegetables	0.01	0.00	0.00	0.00	0.00	0.01	0.01	0.01	0.01	0.01
Tea										
Green Tea	0.00	0.00	0.00	0.00	0.00	0.00	0.00	0.00	0.01	0.01
Black Tea	0.00	0.00	0.00	0.00	0.00	0.00	0.00	0.00	0.01	0.00
Oolong Tea	0.00	0.00	0.00	0.00	0.00	0.00	0.00	0.00	0.01	0.00
Dark Tea	0.00	0.00	0.00	0.00	0.00	0.00	0.00	0.00	0.00	0.00
Fresh Aquatic Products										
Fish	0.01	0.01	0.01	0.01	0.01	0.03	0.02	0.01	0.02	0.02
Crustaceans	0.01	0.01	0.01	0.00	0.00	0.03	0.02	0.02	0.01	0.01
Mollusks	0.01	0.01	0.01	0.01	0.01	0.09	0.05	0.03	0.04	0.04
Fresh Fruits										
Citrus Fruits	0.01	0.01	0.00	0.00	0.01	0.02	0.01	0.01	0.01	0.01
Melons and Gourd Fruits	0.01	0.01	0.01	0.01	0.01	0.03	0.02	0.01	0.01	0.01
Stone Fruits	0.01	0.01	0.01	0.00	0.00	0.02	0.01	0.01	0.01	0.01
Berries and Other Small Fruits	0.01	0.01	0.00	0.00	0.00	0.02	0.01	0.01	0.01	0.01
Tropical and Subtropical Fruits	0.01	0.01	0.00	0.00	0.00	0.02	0.01	0.01	0.01	0.01
Pome Fruits	0.01	0.01	0.01	0.01	0.01	0.02	0.01	0.01	0.01	0.01
Canned Foods										
Cereal-based Canned Foods	0.00	0.00	0.04	0.02	0.04	0.00	0.00	0.04	0.04	0.06
Canned Vegetables and Vegetable Products	0.00	0.07	0.00	0.04	0.03	0.00	0.07	0.00	0.04	0.04
Canned Fruits and Fruit Products	0.00	0.00	0.00	0.10	0.00	0.00	0.00	0.00	0.10	0.00
Canned Meat and Meat Products	0.01	0.01	0.01	0.01	0.01	0.09	0.08	0.07	0.03	0.03
Canned Aquatic Products	0.02	0.01	0.01	0.01	0.01	0.10	0.09	0.08	0.03	0.03
Canned Edible Fungi and Fungal Product	0.00	0.04	0.00	0.04	0.04	0.00	0.05	0.00	0.05	0.04
ΣEDI	0.16	0.23	0.14	0.29	0.20	0.55	0.54	0.35	0.46	0.37
THQ	0.0005	0.0008	0.0005	0.0010	0.0007	0.0018	0.0018	0.0012	0.0015	0.0012
	**C: P50** **×** **P95**					**D: P95** **×** **P95**				
Fresh Vegetables										
Solanaceous Vegetables	0.68	0.48	0.42	0.42	0.45	1.64	1.21	0.78	0.79	0.90
Cruciferous Vegetables	0.08	0.07	0.06	0.10	0.05	0.08	0.12	0.09	0.10	0.10
Cucurbit Vegetables	0.09	0.07	0.06	0.06	0.06	0.24	0.16	0.12	0.12	0.12
Tuber and Root Vegetables	0.73	0.60	0.51	0.54	0.55	1.68	1.49	0.95	1.08	1.11
Aquatic Vegetables	0.07	0.06	0.06	0.05	0.05	0.21	0.17	0.11	0.10	0.12
Leafy Vegetables	0.52	0.49	0.39	0.33	0.34	1.24	0.92	0.71	0.66	0.68
Tea										
Green Tea	0.00	0.01	0.02	0.02	0.02	0.00	0.01	0.04	0.06	0.07
Black Tea	0.00	0.03	0.02	0.01	0.01	0.00	0.03	0.02	0.04	0.03
Oolong Tea	0.00	0.00	0.00	0.01	0.03	0.00	0.00	0.00	0.05	0.03
Dark Tea	0.00	0.00	0.00	0.02	0.04	0.00	0.00	0.00	0.01	0.02
Fresh Aquatic Products										
Fish	1.26	0.74	0.67	0.64	0.58	2.51	2.23	1.43	1.61	1.66
Crustaceans	0.26	0.15	0.12	0.10	0.09	0.62	0.46	0.39	0.29	0.26
Mollusks	0.08	0.05	0.04	0.03	0.03	0.54	0.28	0.21	0.22	0.22
Fresh Fruits										
Citrus Fruits	0.05	0.04	0.02	0.02	0.04	0.11	0.07	0.05	0.04	0.04
Melons and Gourd Fruits	0.09	0.06	0.05	0.04	0.05	0.19	0.14	0.07	0.09	0.09
Stone Fruits	0.05	0.04	0.03	0.02	0.02	0.11	0.07	0.05	0.04	0.04
Berries and Other Small Fruits	0.02	0.01	0.01	0.01	0.01	0.04	0.03	0.02	0.02	0.02
Tropical and Subtropical Fruits	0.03	0.02	0.01	0.01	0.01	0.07	0.04	0.03	0.03	0.03
Pome Fruits	0.18	0.13	0.09	0.08	0.08	0.35	0.22	0.15	0.13	0.13
Canned Foods										
Cereal-based Canned Foods	0.00	0.00	1.19	0.50	1.22	0.00	0.00	1.19	1.21	1.60
Canned Vegetables and Vegetable Products	0.00	3.04	0.00	1.65	1.27	0.00	3.04	0.00	1.65	1.61
Canned Fruits and Fruit Products	0.00	0.00	0.00	52.00	0.00	0.00	0.00	0.00	52.00	0.00
Canned Meat and Meat Products	1.72	1.35	1.31	1.10	0.75	11.45	10.13	8.05	3.67	3.77
Canned Aquatic Products	0.52	0.41	0.40	0.33	0.23	2.92	2.58	2.22	0.93	0.96
Canned Edible Fungi and Fungal Product	0.00	0.29	0.00	0.31	0.28	0.00	0.42	0.00	0.36	0.28
ΣEDI	6.43	8.14	5.48	58.44	6.29	24.01	23.81	16.68	65.29	13.90
THQ	0.0214	0.0271	0.0183	0.1948	0.0210	0.0800	0.0794	0.0556	0.2176	0.0463
RDF	300 ug/kg BW/day									

Note: A: consumption P50 multiplied by Sn pollution level P95; B: consumption P95 multiplied by Sn pollution level P50; C: consumption P50 multiplied by Sn pollution level P95; D: consumption P95 multiplied by Sn pollution level P95.

**Table 4:** Organotin dietary exposure assessment for fresh aquatic products in Zhejiang residents (µg/kg BW/day) (RDF = 0.1 µg/kg BW/day).

Scenario	Age Group	≤6	7–12	13–17	18–59	≥60
Scenario A	ΣEDI	0.04	0.02	0.02	0.02	0.02
	THQ	0.4	0.2	0.2	0.2	0.2
Scenario B	ΣEDI	0.15	0.09	0.07	0.07	0.07
	THQ	1.5	0.9	0.7	0.7	0.7
Scenario C	ΣEDI	1.6	0.94	0.83	0.78	0.7
	THQ	16	9.4	8.3	7.8	7
Scenario D	ΣEDI	3.68	2.96	2.03	2.12	2.14
	THQ	36.8	29.6	20.3	21.2	21.4

Notes: Scenario A: consumption P50× organotin pollution level P50; Scenario B: consumption P95× organotin pollution level P50; Scenario C: consumption P50× organotin pollution level P95; Scenario D: consumption P95× organotin pollution level P95. Organotin concentrations in fish were estimated using a conservative literature-derived organotin-to-total Sn proportion (5.83%) from mollusks [50].

**Table 5:** Monte Carlo estimated percentiles of dietary organotin exposure at the TDI level for fresh aquatic products by age group.

Age Group	Percentile at TDI (%) ^1^	EDI at Percentile (µg/kg BW/day) ^2^	95% CI Lower (%) ^3^	95% CI Upper (%) ^3^
≤6	69.3	0.1	68.4	70.2
7–12	67.7	0.1	66.8	68.6
13–17	65.8	0.1	64.8	66.7
18–59	65	0.1	64.2	65.9
≥60	65.6	0.1	64.6	66.6
total	66.3	0.1	65.4	67.3

Notes:^1^ Percentile of the Monte Carlo simulated exposure distribution at which the EDI reaches or just exceeds the tolerable daily intake (TDI = 0.1 µg/kg BW/day). ^2^ Dietary exposure corresponding to that percentile. ^3^ 95% confidence interval estimated from the Monte Carlo simulation.

## Data Availability

The data presented in this study are available upon request from the corresponding author due to privacy/ethical restrictions.

## Supplementary Materials

The following supporting information can be downloaded at: https://www.mdpi.com/article/10.3390/foods15060982/s1, Table S1: Geographic origin and distribution of food samples collected in Zhejiang Province, China, Table S2: Provincial origin of canned food samples collected in Zhejiang Province, China, Table S3: Distribution of packaging types and sampling site categories across food groups.
