# Supplementary material for: Occurrence of Tin in Foods and Dietary Exposure Assessment in Zhejiang Province, China
Source: Foods. 2026 Mar 10;15(6):982. doi: 10.3390/foods15060982 (PMC13025888; doi:10.3390/foods15060982)
Supplement: Supplementary file 1 [file foods-15-00982-s001.zip › foods-4159406-supplementary.pdf]

Table S1. Geographic origin and distribution of food samples collected in Zhejiang Province, China.

Food category	Zhejiang Province n (%)	Other regions of China n (%)	Imported n (%)	Total (n)
Fresh vegetables	703 (99.6)	3 (0.4)	0 (0.0)	706
Tea	301 (79.6)	76 (20.1)	1 (0.3)	378
Fresh aquatic products	390 (99.5)	1 (0.3)	1 (0.3)	392
Fresh fruits	122 (91.7)	9 (6.8)	2 (1.5)	133
Canned foods	139 (31.7)	284 (64.8)	15 (3.4)	438

Table S2. Provincial origin of canned food samples collected in Zhejiang Province, China.

Province/Region	n
Zhejiang	139
Fujian	64
Guangdong	59
Sichuan	38
Shanghai	36
Shandong	23
Anhui	18
Hebei	10
Henan	10
Jiangsu	9
Hubei	9
Liaoning	3
Taiwan	2
Jiangxi	1
Hunan	1
Hong Kong	1
Imported	15
**Total**	**438**

Note: Canned food samples were sourced from Zhejiang Province and multiple major food-producing regions in China, reflecting the actual market supply structure in Zhejiang Province.

Table S3. Distribution of packaging types and sampling site categories across food groups

Food category	Characteristic	Category	n (%)
Fresh vegetables (n=706)	Packaging	Bulk	705 (99.9)
		Pre-packaged	1 (0.1)
	Sampling site	Farmers' market	458 (64.9)
		Retail store	248 (35.1)
Tea (n=378)	Packaging	Bulk	255 (67.5)
		Pre-packaged	123 (32.5)
	Sampling site	Retail store	258 (68.3)
		Farmers' market	120 (31.7)
Fresh fruits (n=133)	Packaging	Bulk	133 (100.0)
	Sampling site	Retail store	73 (54.9)
		Farmers' market	58 (43.6)
		Street vendor	2 (1.5)
Fresh aquatic products (n=392)	Packaging	Bulk	389 (99.2)
		Pre-packaged	3 (0.8)
	Sampling site	Farmers' market	298 (76.0)
		Retail store	85 (21.7)
		Online store	7 (1.8)

		Aquaculture site	1 (0.3)
		Capture fishery	1 (0.3)
Canned foods (n=438)	Packaging	Pre-packaged	436(99.5)
		Bulk	2 (0.5)
	Sampling site	Retail store	321 (73.3)
		Farmers' market	105 (2.4)
		Online store	11 (2.5)
